# St. Thomas's Hospital Medical School

**Published:** 1906-07-07

**Authors:** 


### ST. THOMAS'S HOSPITAL MEDICAL SCHOOL.

Mr. J. G. Wainwright, the Treasurer, took the chair on Wednesday at the Distribution of Prizes to students, and in introducing Professor Osler, M.D., F.R.S., who made the distribution, referred to him as perhaps the most distinguished physician in the world. In his opening remarks the Chairman mentioned some of the successes attained in all parts of the world by St. Thomas's students, and while referring with pride to the work accomplished by the hospital regretted that though the expenditure of the year had been £57,000, the income had only amounted to £50,000. They hoped soon to have three new wards available and hoped the necessary extra funds would be forthcoming.

The following were the principal awards:

Prizes for the Summer Session, 1905.—Second Year's Students: W. B. Johnson, College Prize, £15 and Certificate of Honour.

Prizes for the Winter Session, 1905-6.—University Scholarships: G. R. Girdlestone, Scholarship, £50, and Certificate of Honour. Second Year's Students: T. E. A. Stowell, The Wm. Tite Scholarship, £25, and Certificate of Honour; W. L. Pink, College Prize, £20, and Certificate of Honour; R. C. Maybury, College Prize, £10, and Certificate of Honour. Third Year's Students: W. B. Johnson, The Peacock Scholarship, £35, and Certificate of Honour;

E. F. Ballard, College Prize, £20, and Certificate of Honour. Fourth Year's Students: R. W. Rix, Second Tenure of Musgrove Scholarship, and Certificate of Honour. Fifth Year's Students: Medicine—(*Seniors*) N. R. Cunningham, £10 and Certificate of Honour; (*Juniors*) H. J. Nightingale, £10 and Certificate of Honour. Surgery—(*Seniors*) C. M. Page, £10 and Certificate of Honour; (*Juniors*) H. J. Nightingale, £10 and Certificate of Honour. Midwifery—(*Seniors*) H. B. Whitehouse, £10 and Certificate of Honour; (*Juniors*) A. C. F. Turner, £10 and Certificate of Honour. Pathology (*Seniors*) H. A. Philpot, £10 (Hadden Prize) and Certificate of Honour; (*Juniors*) A. C. F. Turner, £10 (Hadden Prize) and Certificate of Honour. Pharmacology—(*Seniors*) I. C. Maclean, W. O. Sankey, divide Prize of £10. Certificates of Honour; (*Juniors*) A. C. F. Turner, £10 and Certificate of Honour. Forensic Medicine and Insanity—(*Seniors*) C. M. Page, H. B. Whitehouse, divide Prize of £10, Certificates of Honour; (*Juniors*) A. C. F. Turner, W. H. R. Sutton, divide Prize of £10, Certificates of Honour. Public Health —(*Seniors*) H. Granger, C. M. Page, divide Prize of £10, Certificates of Honour; (*Juniors*) H. G. Bennett, £10 and Certificate of Honour.

Medallists.—(Introduced by the Dean of the Medical School.)—Practical Medicine: H. J. Nightingale, The Mead Medal; H. C. Squires, The Wainwright Prize; H. J. Nightingale, The Seymour Graves Toller Prize; H. G. Bennett, C. M. Page, qualified for Mead Medal, Certificates of Honour; S. Churchill, qualified for Wainwright Prize, Certificate of Honour. Surgery and Surgical Anatomy; C. M. Page, The Cheselden Medal; R. F. Hebbert, qualified for Cheselden Medal, Certificate of Honour. Beaney Scholarship in Surgery, £50: G. R. Footner, L. E. C. Norbury, divide Scholarship. Pathology and Morbid Anatomy: R. C. Jewesbury, The Bristowe Medal. For General Proficiency and Good Conduct: H. J. Nightingale, The Treasurer's Gold Medal.

In presenting the Mead Medal to the winner of the Mead Medal, Mr. H. J. Nightingale, Professor Osler said: "May you be the Mead of the twentieth century, and let me remind you that Dr. Johnson said that no other man he had known had so lived in the sunshine of life as Richard Mead."

